# Interactions between mannose-binding lectin and MASPs during complement activation by the lectin pathway

**DOI:** 10.1016/j.imbio.2006.11.004

**Published:** 2007-06-26

**Authors:** Russell Wallis

**Affiliations:** a Departments of Infection, Immunity and Inflammation and Biochemistry, University of Leicester, Leicester, UK; b MRC Immunochemistry Unit, Department of Biochemistry, University of Oxford, Oxford, UK

**Keywords:** Complement, Immunodeficiency, Innate immunity, Lectin pathway, Protein interactions, CCP, complement control protein, CRD, carbohydrate-recognition domain, CUB domain, domain found in complement component Clr/Cls, Uegf and bone morphogenic protein 1, EGF, epidermal growth factor, MASP, MBL-associated serine protease, MBL, mannose-binding lectin

## Abstract

The lectin pathway of complement performs a key role within the immune system by recognising pathogens through patterns of sugar moieties displayed on their cell surfaces and neutralising them via an antibody-independent reaction cascade. While particularly important during early childhood before the adaptive immune system is established, or when adaptive immunity is compromised, it has a protective function throughout life, neutralising invading pathogens directly and helping to stimulate and direct an effective immune response. Complement activation is initiated when complexes comprising mannose-binding lectin (MBL) or serum ficolins and MBL-associated serine protease-2 (MASP-2) bind to pathogens. Binding induces conformational changes in these complexes, leading to autoactivation of the MASPs, which in turn activate the downstream reaction cascade. A major goal in complement research is to understand the molecular events that trigger complement activation. Over the last few years, structure–function studies have improved our knowledge of the way in which MBL binds to MASPs by defining the portions of these proteins that interact and by solving the structures of key protein fragments. In this review, I will summarise the main findings of these studies and describe current theories to explain how the components combine to initiate the reaction cascade.

## Introduction

The complement cascade is a fundamental component of the immune system, providing protection against invading microorganisms through both antibody-dependent and -independent mechanisms (Porter and Reid, 1978). It also mediates many cellular and humoral interactions within the host, including chemotaxis, phagocytosis, cell adhesion and B-cell differentiation, thereby helping to coordinate and direct an effective immune response (Carroll, 2004). Three different pathways initiate the complement cascade: the classical, alternative and lectin pathways. This review will focus on the biochemical aspects of lectin pathway complement activation, the most recently discovered and probably the most ancient of the three activation pathways (Dodds, 2002).

Similar to the C1 complex of the classical pathway, the initiating complexes of the lectin pathway comprise separate recognition and enzyme components. The recognition components, mannose-binding lectin (MBL) and serum ficolins, bind directly to sugars or *N*-acetyl groups on pathogenic cells and activate three different enzymes, called MBL-associated serine proteases (MASPs-1 to -3), to activate complement (Turner, 1996; Schwaeble et al., 2002). Currently, only MASP-2 has a clearly defined role in complement activation (Thiel et al., 1997; Rossi et al., 2001). It initially cleaves C4 to produce the peptide anaphylatoxin C4a and the C4b fragment, which attaches to the activating surface of the pathogen upon exposure of the highly reactive thioester group. C2 then binds to the immobilised C4b molecule and is also cleaved by MASP-2 to generate C2b and C2a. C2a remains attached to C4b to become the catalytic component of the C3 convertase (C4b2a), the enzyme that catalyses the next step in the reaction pathway (Kerr, 1980). The roles of MASP-1 and MASP-3 are not known. MASP-1 cleaves C2 but not C4, so it might enhance complement activation triggered by lectin–MASP-2 complexes, but cannot initiate activation itself (Chen and Wallis, 2004). The only substrate that has been identified for MASP-3 is insulin-like growth factor-binding protein 5 (Cortesio and Jiang, 2006). However, it is unclear if this is a physiological substrate.

The importance of the lectin pathway to human health is highlighted by immunodeficiencies associated with mutations to MBL (Turner, 1996) and MASP-2 (Stengaard-Pedersen et al., 2003). Individuals homo- or heterozygous for any one of three point mutations in the MBL gene or with mutations to the promoter region are susceptible to a wide range of bacterial, viral and parasitic infections, particularly in early childhood, before adaptive immunity is established (Sumiya et al., 1991) or when the adaptive immune response is compromised, for example, during HIV infection or following chemotherapy (Garred et al., 1997; Neth et al., 2001). The lectin pathway also plays a role in the pathogenesis of inflammatory disorders, such as cystic fibrosis and rheumatoid arthritis, where MBL deficiency is associated with more severe disease (Kilpatrick, 2002). Because of its key protective roles, MBL is a strong candidate for clinical trials to reduce susceptibility to infections or for use as a disease-modifying agent (Summerfield, 2003).

While complement generally has a protective role, under certain circumstances, inappropriate activation causes significant host damage. For example, tissues including heart and kidney are subject to significant complement-mediated cytotoxicity upon reperfusion of oxygenated blood following transient ischaemia (Carroll and Holers, 2005). In several recent studies, the lectin pathway has been directly implicated in triggering such inappropriate activation (Hart et al., 2005; Moller-Kristensen et al., 2005; Walsh et al., 2005). By preventing host-mediated tissue damage, selective lectin pathway inhibitors are likely to have important therapeutic benefits for treatment of common, potentially life-threatening diseases, including ischaemic heart disease, kidney disease and reperfusion injuries. Thus, understanding how complement activation is initiated and regulated is of great interest both from a biochemical perspective and also as a step towards designing therapeutics to modulate the immune response.

## Structural organisation of MBL and MASPs

### Structural organisation of MBL

**Fig. 1:**
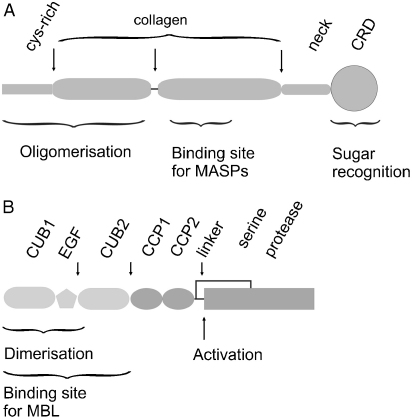
Domain organisation of MBL and MASPs. (A) Structural organisation of MBL. The collagenous domain is shown as two segments, which are separated by the interruption in the Gly–Xaa–Yaa collagen repeat. Arrows show potentially flexible regions. (B) Structural organisation of MASPs. Regions that mediate interactions with MBL are shown in light grey. Regions involved in substrate recognition are shown in dark grey. The position of the cleavage site for zymogen activation is marked by an arrow. The solid line indicates the disulphide bond that links the N- and C-terminal fragments together following activation. Arrows above the figure show potentially flexible regions.

MBL consists of large oligomers assembled from identical polypeptide chains (Drickamer and Taylor, 1993). Three polypeptides assemble into subunits, which subsequently associate to form larger oligomers. Each subunit is composed of an N-terminal cysteine-rich domain followed by a collagenous domain, an *α*-helical coiled coil or neck region and three C-terminal C-type carbohydrate-recognition domains (CRDs) (Fig. 1A). Interchain disulphide bonds link the N-terminal domains of polypeptides together both within and between subunits to stabilise oligomers (Wallis and Drickamer, 1999). Although the compositions of MBL oligomers are generally similar in different species, there are some differences. For example, human MBL comprises dimers to hexamers of subunits of which trimers and tetramers are probably the predominant species (Lu et al., 1990; Teillet et al., 2005), while rat MBL is mainly composed of dimers, trimers and tetramers of subunits (Wallis and Drickamer, 1999).

Heterogeneity in MBL probably arises because of the way in which oligomers assemble within the endoplasmic reticulum of liver cells during biosynthesis. Pulse-chase experiments show that while individual MBL subunits assemble rapidly, the protein matures into the larger oligomeric forms more slowly as it moves to the cell surface for secretion (Heise et al., 2000). A key factor controlling the sizes of oligomers appears to be the pattern of disulphide bonds at the N-terminal ends of subunits. Each polypeptide in human MBL has three cysteine residues within this region that can potentially form disulphide bonds. Certain bonding patterns allow interchain bonds to two additional subunits and are thus compatible with further oligomerisation, whereas other patterns permit linkage to only one subunit, therefore serving as ‘terminating patterns’ (Jensen et al., 2005). Before secretion, any surface-exposed, unpaired cysteine residues are likely to be capped through disulphide bonding to cysteine. This process is necessary because free cysteine residues probably act as retention signals for MBL as they do for many other secreted proteins, so only those oligomers with no exposed cysteines are ready for secretion (Wallis and Drickamer, 1999; Jensen et al., 2005).

Images of MBL obtained by electron microscopy reveal bouquet-like structures, with rod-like collagen stems topped by globular CRDs (Lu et al., 1990). An interruption (Gly–Gln–Gly) in the collagen Gly–Xaa–Yaa repeat within the N-terminal half of the collagenous domain introduces a kink causing the stems to splay apart. Although there is no direct evidence for flexibility at this site in MBL, visualisation of type IV collagen chains by electron microscopy shows some flexible sites, and the positions of these sites correlate with several sequence interruptions (Mohs et al., 2006). While all MBLs have a break in the collagenous domain, some of the ficolins do not (Fujita et al., 2004). Here, the junction between the cysteine-rich domain and the collagenous domain might serve as a flexible hinge. The same region might also be flexible in MBL, although no structural information is available for this region. The junction between the collagen triple helix and the α-helical neck region represents a third potentially flexible region in MBL. Within the collagenous domain, each of the three polypeptide chains is staggered by one residue whereas all three chains become aligned in the *α* helical coiled coil, so at the junction there must be a change in the alignment of the polypeptide chains. Corresponding regions in type I and II class A macrophage scavenger receptors are highly flexible (Resnick et al., 1996), so this junction probably also allows some degree of movement in MBL. One or more of the three potentially flexible regions of MBL are probably very important to allow the conformational changes that induce complex activation (see below). Flexibility at the junction between the collagen-like domain and the neck probably enables the CRDs to orient as the molecule docks onto a bacterial cell surface. The subsequent conformational changes generated at the other end of the molecule cause the subunits to realign, providing the trigger for MASP activation.

MBLs recognise foreign cells via multivalent interactions with the carbohydrate epitopes commonly found on pathogens. Each CRD has a single binding site for monosaccharides such as mannose, fucose or *N*-acetylglucosamine, which occur only rarely at the terminal positions of mammalian oligosaccharides on glycoproteins and glycolipids but are present in high-density arrays on many bacterial, fungal and parasitic cells (Drickamer, 1993). The sugar-binding site is localised around one of two Ca^2+^ sites (Weis et al., 1992). Equatorial hydroxyl groups at the 3- and 4-OH positions of the sugar residue serve as coordination ligands for the Ca^2+^. Additional coordination ligands are provided by asparagine and glutamic acid residues in the CRD that also form hydrogen bonds with the 3- and 4-OH groups of the sugar residue. The limited nature of the contacts between sugar and protein enables MBL to recognise a broad range of target ligands. Thus, sugars which have equatorial OH-groups equivalent to those of the 3- and 4-OH groups of mannose are ligands. However, galactose and related sugars that have an axial 4-OH group are not recognised by MBL. The affinities of CRDs for monosaccharides are typically weak (∼1 mM) (Iobst et al., 1994). Consequently, multiple CRD–sugar interactions involving more than one MBL subunit are necessary for stable binding of MBL to pathogens and also for complement activation.

### Structural organisation of MASPs and MAp19

MASPs are homologues of C1r and C1 s of the classical pathway. They all have the same modular organisation consisting of two domains found in complement component Clr/Cls, Uegf and bone morphogenic protein 1 (CUB domains), separated by a Ca^2+^-binding epidermal growth factor (EGF)-like domain and followed by two complement control protein (CCP) modules and a C-terminal serine protease domain (Schwaeble et al., 2002) (Fig. 1B). MASPs-1 and -3 are encoded by a common gene through alternative splicing. They have identical N-terminal domains but different linker regions and serine protease domains. A small non-enzymatic protein called MAp19 or sMAP also associates with lectin–MASP complexes. MAp19 is an alternatively spliced product of the MASP-2 gene, consisting of the N-terminal CUB1 and EGF-like domains of MASP-2.

MASPs normally circulate as zymogens. However, when lectin–MASP complexes bind to target epitopes on pathogens, MASPs-1 and -2 activate through autolysis at a single site within the short linker region between the CCP-2 module and serine protease domain. The active protease domain remains attached to the N-terminal fragment through a single disulphide bond. MASP-3 is also activated through cleavage of the linker region. However, the zymogen cannot autoactivate, so is probably activated through the action of an unidentified serum protease (Zundel et al., 2004).

**Fig. 2:**
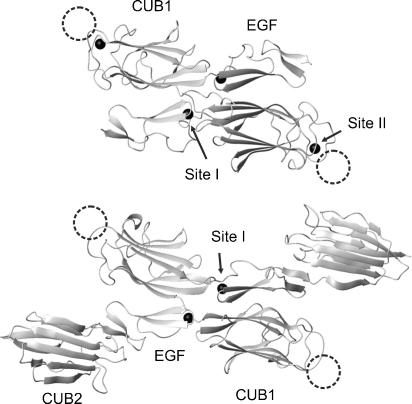
Structures of human MAp19 (top) and the CUB1–EGF–CUB2 fragment of rat MASP-2 (bottom). The probable location of the high-affinity binding sites for MBL subunits is shown by circles, the diameters of which roughly correspond to that of a collagen triple helix. Additional low-affinity sites are probably located on each of the CUB2 domains.

Recently, the structures of the rat CUB1–EGF–CUB2 fragment and human MAP-19 have been solved (Feinberg et al., 2003; Gregory et al., 2004) (Fig. 2). In both structures, lateral association of the CUB1 and EGF-like domains about a 2-fold symmetry axis creates a relatively compact dimer arranged in a head-to-tail configuration with extensive interactions between the CUB1 and the EGF-like domains in the dimer interface. The structures of fragments of MASPs comprising the CCP-2-serine protease domain and CCP-1–CCP-2-serine protease domain have also been solved (Harmat et al., 2004; Gál et al., 2005). Interestingly, these structures reveal that the comparable substrate specificities of MASP-2 and C1s, of the classical pathway, are realised through different sets of enzyme–substrate interactions. Comparison of the active and zymogen forms of MASP-2 has provided novel structural insight into MASP-2 activation. These data are reviewed by Peter Gál and colleagues in this edition of Immunobiology, and will not be covered here (Gál et al., 2007).

While the CUB1–EGF and CCP-1–CCP-2 junctions are probably quasi-rigid, structural and biochemical evidence suggests that the EGF–CUB2, CUB2–CCP-1 and CCP-2–serine protease junctions are all potentially flexible (Feinberg et al., 2003; Gaboriaud et al., 2004; Gal et al., 2005). These sites probably allow the conformational changes required to bring the two serine protease domains into close apposition during activation (Fig. 1).

## Interactions between MBL and MASPs

MBL binds to MASPs-1, -2 and -3 with affinities in the nM range (3.2 and 2.6 nM for human MBL–MASP-1 and MBL–MASP-2, respectively) (Wallis and Dodd, 2000; Thielens et al., 2001). Binding is mediated through a portion of the collagenous domain of MBL and the N-terminal domains of the MASP. All three N-terminal MASP domains (CUB1–EGF–CUB2) are necessary and sufficient to reproduce the binding properties of the full-length proteins. The CUB1–EGF domains also bind to MBL, but with lower affinity than the N-terminal three domains and full-size proteins. Consequently, MAp19 binds to MBL ∼5-fold and ∼10 more weakly than full-size MASP-2 in human and rat complexes, respectively.

### Stoichiometry of MBL–MASP complexes

Different MBL oligomers are able to activate complement, but with different activities (Wallis and Drickamer, 1999). In rat MBL, purified trimers and tetramers of MBL subunits have the highest activities (1.18±0.23 and 0.95±0.05 relative to wild-type protein), followed by MBL dimers (0.24±0.06), while single subunits have no detectable activity (<0.01) (Wallis and Drickamer, 1999). The reason that MBL dimers, trimers and tetramers are all functional while single MBL subunits are not is due largely to the stoichiometry of complexes, in which MASP dimers have binding sites for at least two MBL subunits (Chen and Wallis, 2001). The most likely arrangement (see below) is where each protomer of the MASP dimer contains a high-affinity binding site for one MBL subunit. Thus, MBL dimers bind stably to single MASP dimers whereas trimers and tetramers can bind up to two MASP dimers, although 1:1 complexes appear to be more stable than 1:2 complexes (Chen and Wallis, 2001; Teillet et al., 2005). Consequently, dimers, trimers and tetramers can bind with high affinity to at least one MASP dimer. Although single MBL subunits also bind to MASPs, the affinities are ∼1000-fold lower than the larger MBL oligomers, hence their low biological activities (Chen and Wallis, 2001).

Differences in the abilities of MBL oligomers to activate complement are probably due, at least in part, to their different affinities for MASPs. The CUB1–EGF–CUB2 domains of rat MASP-2 bind to MBL trimers and tetramers with comparable affinities (affinities relative to wild-type MBL: 1.0±0.5 and 1.1±0.5 for MASP-2 and 1.0±0.5 and 1.3±0.3 for MASP-1, respectively), while MBL dimers bind more weakly (0.79±0.21 for MASP-2 and 0.20±0.08 for MASP-1) (Chen and Wallis, 2001). The reason that MBL trimers and tetramers bind to MASPs more tightly than MBL dimers is not known. However, one possibility is that each protomer of the MASP dimer contains an extra low-affinity binding site for an MBL subunit in addition to the high-affinity site already described, giving a total of four subunit-binding sites (two high- and two low-affinity sites) per MASP dimer (Teillet et al., 2006). Consequently, MBL trimers and tetramers would bind to one or both of the low-affinity sites as well as to both of the high-affinity sites.

While the stoichiometry of MBL oligomers binding to each MASP has been determined, the composition of circulating MBL–MASP complexes is still unknown. MASPs-1 and -2 do not interact with each other in the absence of MBL (Chen and Wallis, 2001). However, the larger MBL oligomers can potentially bind more than one MASP, so in vivo these oligomers might circulate bound to two different MASPs or one MASP together with MAp19. Analysis of human serum tentatively supports this suggestion, because larger MBL oligomers appear to associate with MASP-2 and MASP-3, while smaller MBL oligomers bind to MAp19 and MASP-1, although the composition of complexes is not known (Dahl et al., 2001). Thus, it is possible that certain complexes form preferentially because they are stabilised through cooperative interactions between components.

### Ca^2+^ dependence of MBL–MASP interactions

The interaction between MBL and MASPs is Ca^2+^ dependent. The critical Ca^2+^ sites are probably located within the CUB1–EGF fragment of the MASPs. In both of the crystal structures available, a single Ca^2+^ (site I) is bound near the N-terminal end of the EGF-like domain of each protomer, with 4 and 6 conserved coordination ligands contributed by the protein in the CUB1–EGF–CUB2 and Map19 structures, respectively. While dimerisation occurs even in the presence of chelators and is thus probably Ca^2+^ independent, several residues, which lie in or near the Ca^2+^ site, participate in interactions with the CUB1 domain of the adjacent protomer, stabilising the dimer.

**Fig. 3:**
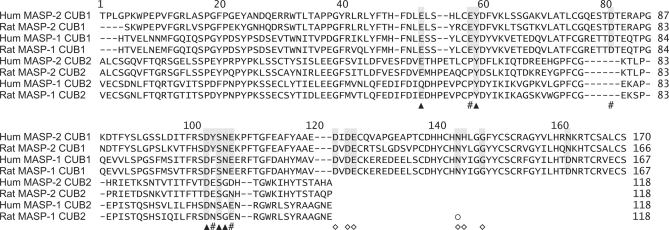
Aligned sequences of the CUB and EGF domains of MASP-2 and MASP-1/3. Residues providing coordination ligands for Ca^2+^ in the EGF (site I) and CUB1 domains (site II) of human Map19 are marked by (◊) and (▴), respectively. In the rat CUB1–EGF–CUB2 structure the only coordination ligands observed in the structure are provided by main chain carbonyl oxygen atoms of Val120 and Tyr140 and the side chains of Asp119 and Asn139. Residues proposed to form part of the binding site for MBL in the CUB1 domain of human Map19 are indicated by (#). The position of the asparagine residue that is hydroxylated on the *β* carbon within the EGF-like domain of rat MBL is marked by (○). Residues at key positions that are identical to those in human MASP-2 are shaded.

In the structure of human MAp19 (and also in C1s (Gregory et al., 2003)), a second Ca^2+^ (site II) is bound to the distal end of each CUB1 module, through five protein ligands and a water molecule (Gregory et al., 2004). Significantly, the residues that provide these coordination ligands are completely conserved in rat MASP-2 (Fig. 3), although some of these residues are disordered in the rat structure and no Ca^2+^ is seen (Feinberg et al., 2003). Given the sequence identity between the human and rat proteins, it is likely that a Ca^2+^ does normally bind to the CUB1 module of rat MASP-2 in solution, but the site was not occupied to a significant extent in the crystal structure, probably because of chelation by tartrate, which was a component of the crystallisation buffer. As site I is occupied in the rat structure but site II is not, the affinity of Ca^2+^ for site II in the free MASP molecule is probably weaker than for site I. Interestingly, sequence alignments reveal that four out of the five residues that provide coordination ligands for Ca^2+^ in CUB1 of Map19 are also present in equivalent positions in CUB2 (Fig. 3), implying that this CUB domain might also bind Ca^2+^.

Analysis by isothermal titration calorimetry shows that Ca^2+^ binds to rat MASP-2 CUB1–EGF–CUB2 fragment with a Kd of 6.3±2.6 μM (Feinberg et al., 2003). Additional, weaker binding was also detected; however, this was concomitant with precipitation of the protein at higher concentrations of Ca^2+^. The Ca^2+^ dependence of MBL binding by the CUB1–EGF–CUB2 fragment was also 6.3 μM, measured by analytical ultracentrifugation. Thus, the interaction between MASP-2 and MBL might depend on the single Ca^2+^ measured by calorimetry. However, it is not clear whether Ca^2+^ was binding to site I or II in these experiments and the affinity for Ca^2+^ at one or both sites might change when MASP-2 binds to MBL, so further studies are required to clarify the roles of Ca^2+^ in the MASPs.

Interestingly, additional evidence about the Ca^2+^ dependence of MBL–MASP binding comes from recent studies of MASP-2 deficiency (Stengaard-Pedersen et al., 2003; Sorensen et al., 2005). The only individual currently described with this condition has severe immunodeficiency and autoimmune reactions. The protein encoded by the MASP-2 gene contains a single mutation at position 105 in the mature polypeptide, in which the aspartic acid of the wild-type sequence is replaced by glycine. Serum MASP-2 levels are lower than normal and the protein that is present fails to associate with MBL or ficolins. Normally, Asp105 provides one of the ligands for Ca^2+^ at site II in the CUB1 domain (Fig. 3), so a likely explanation for the observed phenotype is that the mutation reduces the ability of the MASP to bind Ca^2+^ at site II, suggesting that this Ca^2+^ is critical for MBL binding. Confirmation of this theory, however, awaits additional studies.

Site-directed mutagenesis of MAp19 indicates that the high-affinity binding site for MBL is probably located at the distal end of the CUB1 domain (Fig. 2), near Ca^2+^ site II (Gregory et al., 2004). The head-to-tail arrangement of protomers in the MASP dimer means that each protomer is able to bind to a separate MBL subunit through equivalent interactions. Significantly, residues identified as putative contacts for MASP-2 are conserved in rat MASP-2 and are also largely present in human and rat MASP-1 (Fig. 3). Thus, it is likely that the MBL-binding sites are equivalent in the human and rat proteins and that all three MASPs bind to MBL through at least some comparable contacts.

### MASP binding to the collagenous domain of MBL

**Fig. 4:**
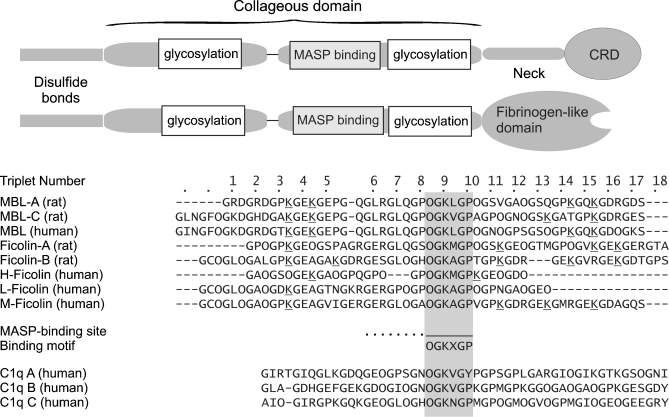
Proposed location of MASP-binding sites within the collagenous domain of MBL and ficolins. Top, domain organisation of MBLs and ficolins highlighting the positions of the MASP binding sites and potential glycosylation sites. Bottom, aligned sequences of human and rat MBLs and ficolins. The consensus motif for MASP binding present in MBLs and ficolins is shaded. Positions of hydroxyproline (O) and glycosylated hydroxylysine (K) residues are based on the sequences of rat MBLs, in which all proline and lysine residues at the third position of the Gly–Xaa–Yaa are at least partially modified, with the exception of the proline residue in repeat 5, which was completely unmodified by Edman degradation (Wallis and Drickamer, 1997; Wallis and Drickamer, 1999).

The binding site for MASPs is located within the collagen-like domain of MBL, beyond the interruption in the Gly–Xaa–Yaa collagen repeat (Wallis et al., 2004). Sequence alignment of MBLs and ficolins from a variety of species reveals the presence of a common MASP-binding motif: Hyp–Gly–Lys–Xaa–Gly–Pro, where Hyp is 4-hydroxyproline and Xaa is an aliphatic amino acid residue other than glycine (Fig. 4). Replacement of the lysine and aliphatic residues (leucine in rat MBL) with proline and hydroxyproline (stabilising substitutions in collagen) in rat MBL completely abolishes its ability to bind to MASPs or activate complement (Wallis et al., 2004). While one or both of these residues are essential for binding to all three MASPs, adjacent residues appear to modulate binding but to different extents in MASP-1/-3 and MASP-2. Thus, while the MASP binding sites on MBL overlap, they are probably not identical. Interestingly, although the MASP-binding sites themselves do not contain glycosylated 5-hydroxylysine residues, they are flanked by glycosylated residues, suggesting that one function of glucosylgalactosyl-5-hydroxylysine in collagen might be to prevent non-specific interactions with other macromolecules.

**Fig. 5:**
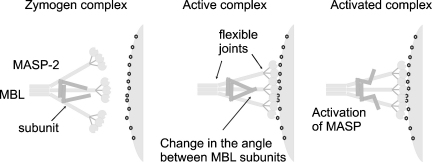
Model of MASP activation by MBL. Left, MASP dimer bridges two MBL subunits (the outermost subunits in this example) through equivalent interactions between each protomer of the MASP and the collagen-like domain of separate MBL subunits. Middle, binding to a bacterial surface induces a change in the angle between MBL subunits, leading to a change in the conformation of the MASP, increasing the likelihood of reciprocal cleavage of the linker region of one MASP protomer by the protease domain of the other. Right, the protease is then locked into the activated conformation and is able to cleave and activate its downstream substrates.

By combining the available structural and biophysical data, a model can be created of a minimal functional MBL–MASP complex in which each MASP dimer bridges two MBL subunits, through identical interactions between each protomer of the dimer and the collagen-like domain of a separate MBL subunit. This binding arrangement allows the CCP and serine protease domains of the MASP to occupy the space in between MBL subunits in the middle of the cone-shaped MBL oligomer, with enough room to permit conformational changes required to activate the protease upon binding of MBL to a cell surface (Fig. 5) (Feinberg et al., 2003). The extra subunits of MBL trimers and tetramers probably also bind to the MASP through a second lower affinity binding site located on the CUB2 domain of each protomer.

The lectin and classical pathways of complement are closely related with respect to both the structures and functions of their components (Wallis, 2002a; Gaboriaud et al., 2004; Sim and Tsiftsoglou, 2004). Indeed, in vitro studies have shown that larger MBL oligomers bind and activate C1r2C1s2 complexes, the protease components of the classical pathway and homologues of MASPs (Lu et al., 1990). Although not thought to occur in vivo, these interactions raise the possibility that MBL and C1q bind and activate their respective proteases through somewhat comparable mechanisms. While mammalian C1q is a heteroligomer, assembled from three different polypeptides and MBL is homooligomeric, alignment reveals that sequences akin to MASP-binding sites occur at equivalent positions in all three chains of human C1q (Fig. 4). These regions might represent binding sites for C1r2C1s2 complexes.

## MASP activation by MBL

Kinetic studies have shown that MBL activates MASP-2 by increasing the rate of autocatalysis when it binds to an activating surface (Chen and Wallis, 2004). Although relatively little is known about the conformational changes that follow pathogen recognition, no significant changes occur upon sugar binding by isolated CRDs or to the neck and CRD fragments of MBL (Weis et al., 1992; Ng et al., 2002), so a mechanism in which the signal is transmitted to the MASP through individual subunits can be largely ruled out. The most likely activation mechanism is where binding of MBL–MASP complexes to a bacterial surface induces a change in the relative arrangement of MBL subunits. As MASPs span MBL subunits by binding to rod-like collagenous stems that are flanked by hinge-like flexible joints, engagement of a cell surface by two or more trimeric lectin heads probably imparts changes to these joints, which are transmitted to the bound protease (Fig. 5). Subsequent conformational changes at the flexible regions of the MASP align the serine protease domains so that the catalytic site of one protomer can access the linker region of its partner. Reciprocal cleavages of the MASP polypeptides fix the protease domains in the active conformation, enabling recognition and activation of downstream substrates to initiate the reaction cascade. Details of such mechanisms must await further structural analysis of MBP–MASP complexes.

### MBL activation and immunodeficiency

Three separate mutations in the gene encoding human MBL are each associated with a common immunodeficiency (Turner, 1996). The resulting amino acid substitutions in MBL (Arg32→Cys, Gly35→Asp and Gly37→Glu) are all located within the N-terminal portion of the collagenous domain, before the interruption in the Gly–Xaa–Yaa repeat. Originally, immunodeficiency was believed to be caused by low levels of MBL in serum (Super et al., 1989; Lipscombe et al., 1992). However, recent work has shown that serum MBL levels are typically only slightly reduced in individuals with variant alleles, but that the resulting MBLs have reduced abilities to activate complement (Garred et al., 2003). The confusion regarding the underlying cause of the immunodeficiency probably arose because the antibodies traditionally used to capture MBL from serum preferentially recognise the larger MBL oligomers associated with wild-type MBL alleles, while the smaller oligomers present in the serum of individuals with variant alleles are relatively poorly detected. Although not providing a reliable measure of MBL serum concentrations, these antibody-based assays are nevertheless still very useful for disease association studies, because the amounts of MBL detected in plasma samples appear to correlate closely with their abilities to activate the lectin pathway. Nevertheless, use of the terms ‘MBL levels’ and ‘MBL concentrations’ to describe the results of such assays is misleading. In particular, because MBL is believed to be an opsonin in addition to its role in complement activation, and the effects of the MBL mutations on this and other functions is not known.

Studies using human and rat recombinant MBLs to investigate the molecular basis of the disorders reveal that each mutation causes a change in the composition of MBL oligomers in the encoded protein, resulting in fewer of the larger oligomers and more of the smaller oligomers (Super et al., 1992; Wallis and Cheng, 1999; Larsen et al., 2004). In the Arg→Cys variant, the defect is probably caused by adventitious disulphide bond formation, involving the introduced cysteine residue, which impairs assembly of subunits during biosynthesis. The consequent reduction in the ability of rat MBL containing the equivalent mutation to activate complement can be explained entirely by the change in the distribution of oligomers, the smaller oligomers (predominantly monomers and dimers of subunits) having lower intrinsic activities than the larger oligomers (trimers and tetramers of subunits) (Wallis and Cheng, 1999).

Defective oligomerisation in the glycine variants is caused by disruption to the collagen triple helix through incorporation of the bulky acidic side chains. Studies using synthetic collagen peptides have shown that Gly→Asp/Glu substitutions are highly destabilising to helix formation (Persikov et al., 2004). When introduced into rat MBL, these mutations reduce the melting temperature of the collagenous domain from 47 to 39 °C (Wallis et al., 2004). Although, subunits still associate to some extent during biosynthesis, fewer of the larger oligomers are formed. Consequently, the defect can partly be explained by defective oligomerisation (Wallis and Cheng, 1999). Nevertheless, activities of recombinant MBLs containing equivalent mutations are still lower than would be expected, even accounting for differences in the composition of oligomers between variant and wild-type proteins. Here, the additional defect is caused mainly by failure of variant MBL–MASP complexes to activate upon binding to a target carbohydrate ligand (Wallis et al., 2005). Decoupling of carbohydrate binding and MASP activation by the variant MBLs is probably caused by increased flexibility in the N-terminal part of the collagenous domain, preventing the conformational changes that trigger MASP activation.

All three mutations to the collagenous domain of MBL have dominant or partially dominant phenotypes. The basis of these phenotypes has also been investigated in recombinant systems by co-expressing wild-type and variant MBL polypeptides in the same cell line (Wallis, 2002b). These studies show that MBLs are secreted almost exclusively as hetero-oligomers containing wild-type and variant polypeptides, which are defective in activating the complement cascade. Analogous dominant-negative phenotypes would also explain the immunodeficient phenotypes of individuals heterozygous for variant MBL alleles.

Studies using recombinant proteins are extremely useful for helping us understand the molecular defects associated with the variant MBL alleles. However, the situation in vivo is more complex because interactions with other molecules can alter the make-up of circulating complexes. For example, MBL oligomers assembled from variant alleles are more susceptible to proteolysis by matrix metalloproteinases than wild-type MBL (Butler et al., 2002). Proteolysis in vivo might reduce the concentration of circulating protein and also alter the composition of oligomers in the serum. Differences in the rates of biosynthesis might also affect the levels of proteins in serum. Indeed, MBL containing either of the glycine mutations is secreted more slowly than wild-type MBL due to changes during biosynthesis, which might contribute to the somewhat reduced MBL levels in vivo (Heise et al., 2000).

## Concluding remarks

In conclusion, considerable progress has been made in characterising the interactions between MBL and MASPs through a combination of structural and biochemical analysis of protein fragments and molecular biological approaches. By combining the available data, models of MBL–MASP complexes have been generated and will be further refined by experimentation. Nevertheless, the molecular details of the interaction mechanisms require further structural studies of MBL–MASP complexes. Given the heterogeneity and probable flexibility of MBL and MASPs, crystallographic analysis of full size complexes is likely to be extremely challenging, so the use of smaller complexes assembled from protein fragments might be the best way to proceed.

The ultimate objective of this research requires understanding of the structural changes that occur upon activation of the MBL–MASP complex. A major drawback is that complement activation requires an activating surface such as a bacterial cell wall, making structural analysis of complexes very difficult to achieve. One strategy that we are currently using to circumvent this problem is to trap complexes in different activation states using MBLs or MASPs containing mutations at activation-sensitive sites. In this way it should be possible to analyse the activation process, even in the absence of a carbohydrate target. Hopefully, future structural and functional analysis of suitable complexes will provide novel insights into the activation mechanism.
